# Supplementary material for: Public health implications of satellite-detected widespread damage to WASH infrastructure in the Gaza Strip
Source: PLOS Glob Public Health. 2025 Feb 10;5(2):e0004221. doi: 10.1371/journal.pgph.0004221 (PMC11809885; doi:10.1371/journal.pgph.0004221)
Supplement: S1 Appendix — (DOCX) [file pgph.0004221.s001.docx]

S1 Appendix: Water availability and water quality reports in the Gaza Strip

Poor baseline clean water access and water quality in the Gaza Strip prior to October 7, 2023

Prior to the escalation of hostilities on October 7, 2023, the Gaza Strip had four main freshwater sources: “the Coastal Aquifer (accessed via approximately 300 groundwater wells), desalinated water supplied by the three short term low volume [STLV] desalination plants, piped water from the Israeli company Mekorot, and UNRWA and private sources (municipal and private vendors operated small-scale desalination units supplied from groundwater wells, some powered by solar PV, and water tanker trucks)” (UNEP, 2024). In terms of proportionality between water sources prior to October 7, 2023, groundwater extraction accounted for 81% of the total water available to the Gaza Strip population, while approximately 12% was shipped via pipeline by Mekorot, the Israeli state-owned national water company; three seawater desalination plants produced 3%, while municipal desalination plants provided another 3%, based on an April 2022 Palestinian Water Authority (PWA) report on 2021 data (Oxfam, 2024c). This is corroborated by another data source, which stated that the main water source was groundwater, with about 20% coming from desalination plants and cross-border pipelines (IPC, 2024). UNEP stated that water availability in the Gaza Strip prior to October 7, 2023, was 85 liters per person per day (UNEP, 2024).

Amount of water available to individuals on a daily basis since the escalation of hostilities is lower than international minimum standards

Water availability in the Gaza Strip since the escalation of hostilities is well below the Sphere Humanitarian Minimum Standard of 15 liters per person per day (Sphere, 2018) and may very well be below 7.5 liters per person per day, which is the minimum standard for a limited period in an acute phase of drought (Sphere, 2018). In some cases, water availability in the enclave may be less than the estimated minimum amount needed for survival, which is 3 liters per person per day (UNICEF, 2023).

Bulk water pipelines from Israel into the Gaza Strip arrive via three main connection points: a northern connection in Al Mintar, a middle connection in Bani Saeed, and a southern connection in Bani Suheila. Data from UNEP and the WASH Cluster showed the average daily flow in March 2024 between these three pipelines was roughly 12,000 m^3^/day, which represents less than 25% of the daily flow prior to October 7, 2023 (UNEP, 2024). However, updated data for April and May 2024 provided by the PWA and the WASH Cluster showed an increase of water supplied via these pipelines, with an estimated daily flow of roughly 32,400 m^3^/day, a 170% increase over March 2024 (UNEP, 2024). This updated flow data still only represents roughly 60% of the supply capacity via these pipelines (UNEP, 2024).

Local Palestinian water authorities often must repair facilities that have sustained damage due to the conflict, but facilities can also be taken offline by local authorities due to the lack of access of fuel to power such facilities, which might also rely in part on solar power. A lack of access to fuel perpetuates a vicious cycle of access to clean water because WASH facilities intended to purify and distribute clean water are no longer able to do so, and informal means of disposing of wastewater further pollute access to traditional sources of clean water obtained from the Coastal Aquifer. A lack of fuel prevented desalination plants from operating at their full capacity, limited water pumps from delivering water to households, curtailed water trucking, and caused wastewater treatment plants to become inoperable, “leading to wastewater emptying into the sea, further polluting the coastal aquifer” (Ahmed, 2023).
