# Supplementary material for: Public health implications of satellite-detected widespread damage to WASH infrastructure in the Gaza Strip
Source: PLOS Glob Public Health. 2025 Feb 10;5(2):e0004221. doi: 10.1371/journal.pgph.0004221 (PMC11809885; doi:10.1371/journal.pgph.0004221)
Supplement: S2 Appendix — (DOCX) [file pgph.0004221.s002.docx]

S2 Appendix: Third-party organization WASH damage assessments

A March 2024 IPC publication referred to February 2024 Gaza Strip WASH infrastructure data from the Global WASH Cluster, which contained a total of 592 points (470 of which were categorized as “water facility” and 122 of which were categorized as “sewage facility”) (IPC, 2024). According to analysis by the Global WASH Cluster, of the 470 water facility points identified in the dataset, 56% of the facilities were damaged to some extent (n=263) (IPC, 2024). Of the 122 sewage facility points identified in the dataset, 56% of the facilities were damaged to some extent (n=68) (IPC, 2024). Summary statistics of the Global WASH Cluster analysis are shown in Table S2.1.

**Global WASH Cluster damage analysis results**

|  | **Percent of total water facilities analyzed** | **Percent of total sewage facilities analyzed** |
| --- | --- | --- |
| Damaged to some extent | 56% (n=263) | 56% (n=68) |
| Possibly damaged | 11% (n=51) | 13% (n=16) |
| No visible damage | 33% (n=156) | 31% (n=38) |
| **TOTALS** | **100% (n=470)** | **100% (n=122)** |

**Table S2.1.** Results of February 2024 WASH infrastructure damage analysis by the Global WASH Cluster.

According to a January 2024 World Bank/Ipsos Gaza Damage Assessment Bi-weekly Report (6th report, dated January 26, 2024), approximately 57% of WASH facilities analyzed were either damaged or destroyed (World Bank & Ipsos, 2024). Also, according to the report, analysis by a separate organization, UNOSAT (United Nations Satellite Centre), from January 2024 found that averaging WASH damage assessments from all five governorates in the Gaza Strip resulted in 55% of WASH facilities assessed as either damaged or destroyed (World Bank & Ipsos, 2024). (A UN OCHA Flash Update dated January 25, 2024, breaks down the numbers by governorate (UN OCHA, 2024a). The UN also appropriately stated that the absence of visible damage to WASH infrastructure in satellite imagery does not guarantee functionality.)

Finally, a May 2024 analysis by the BBC that utilized “a list of locations provided by Gaza's Coastal Municipalities Water Utility (CMWU)” stated: “Of the 603 water facilities we analysed, 53% appeared to have been damaged or destroyed since 7 October [2023]” (Devlin, Ahmed, Palumbo, 2024).

Takeaways

Taken together, these independent analyses consistently demonstrate that roughly half of the WASH infrastructure in the Gaza Strip has been damaged or destroyed. But even though roughly half of the WASH sites analyzed did not show apparent signs of damage, a March 2024 summary note jointly published by The World Bank, EU, and UN entitled “Gaza Strip Interim Damage Assessment” stated that, at the time, water production capacity was estimated to be less than 5% of the “usual output” (World Bank, EU, UN, 2024).

Journalism outlets, such as the BBC in a May 2024 article (Devlin, Ahmed, Palumbo, 2024), have conducted research quantifying damage to WASH infrastructure in the Gaza Strip, but the nature of news articles and the need to draw and keep an audience from the general public does not allow space for an in-depth, detail-oriented, comprehensively transparent and replicable methodology. While the BBC is a reputable news source and its results can be safely trusted, not all journalism outlets adhere to a common standard for accuracy, so there exists a currently unmet need to independently replicate results borne out in the pages of mass media in a transparent, replicable way has yet to be published in the academic literature.

Additionally, some internal reports that studied WASH infrastructure damage assessment in the Gaza Strip have surfaced from international development organizations privately. This lack of access to the broader academic and scientific community precludes the ability for researchers to replicate results. While this proprietary analytic work is important, not having open access or a transparent methodology is a shortcoming of this work. This study aims to help fill that gap by conducting transparent, replicable research (on an open access basis) on WASH infrastructure damage assessment in the Gaza Strip since October 7, 2023. (A more recent WASH infrastructure damage assessment by UNOSAT from June 2024 found that roughly two thirds of WASH infrastructure had been damaged or destroyed, but we were unable to access this non-public report, which likely includes analysis beyond our study period, which ended on February 22, 2024. Given contemporaneous data and the continuation of the conflict, we would expect to see the number of damaged or destroyed WASH facilities increase over time. Additionally, on August 23, 2024, American news organization CNN reported that 70% of WASH facilities had been damaged or destroyed, based on a July 24 WASH Cluster statement which cited analysis from UNOSAT (Stockwell, Noor Haq, 2024).)
