# Supplementary material for: Public health implications of satellite-detected widespread damage to WASH infrastructure in the Gaza Strip
Source: PLOS Glob Public Health. 2025 Feb 10;5(2):e0004221. doi: 10.1371/journal.pgph.0004221 (PMC11809885; doi:10.1371/journal.pgph.0004221)
Supplement: Attachment — Submitted filename: Perlman-Collins-Van Den Hoek - response to reviewers.pdf [file pgph.0004221.s003.pdf]

November 16, 2024

PLOS Global Public Health
1265 Battery St., Ste. 200
San Francisco, CA 94111

SUBJ: Revisions memo for PGPH-D-24-02192

Dear Dr. Nancy Angeline Gnanaselvam,

Thank you very much for the thoughtful and helpful revisions and comments regarding our original research article entitled "Public health implications of satellite-detected widespread damage to WASH infrastructure in the Gaza Strip." This memo details the revisions we have made.

Replies to PLOS GPH Academic Editor

1. The Academic Editor requested that we remove instances of the copyright symbol from the text on pp. 11-14. We have removed these symbols from the text.

2. The Academic Editor requested source data information for basemaps and accompanying governorate border vector data shapefiles. We have added data source credits to the images and the captions credits for the border shapefiles (governorate boundaries). All geographic data we used complies with your CC-BY 4.0 license. We are not publishing any copyrighted maps or GIS data. The only copyrighted material used in this paper is the satellite imagery provided by Planet Labs PBC; the company granted us permission to publish the imagery they supplied to us for use in academic publications. We have cited the imagery appropriately per their guidelines, using the company's preferred formatting in the images themselves.

3. The Academic Editor requested that we add text discussing ethical approval and the ethics of obtaining the data and conducting the analysis. We have added a Data Ethics Statement at the end of the manuscript, addressing the ethical points you raised.

4. The Academic Editor requested that we upload images to PACE. We have uploaded all images to PACE and successfully received approval on all images.

Revisions from Reviewer #1

The abstract starts with '..Hamas terrorist attacks of October 7, 2023' and I wonder about the relevance of that statement in the overall objective of the paper. Nowhere else in the paper is a history of the conflict or the events leading up to the point discussed in detail, so I wonder why this statement is required in the abstract. In line with what the rest of the paper explores [and does so very well], the statement could just be 'Israel's violence in Gaza in the current offensive' or something on those lines. This statement infers the blame on Hamas - a point of debate which I'm sure the authors are not intending for the paper to explore.

Thank you for this helpful feedback. We have updated the verbiage used in the abstract to reflect your feedback.

Given the nature of the well documented destruction, it would be appropriate in the discussion to add the current international law regarding destruction of water related infrastructure. Amnesty has reports etc. I believe this will add more context to the destruction that has been documented in the paper.

We agree and have added a new subsection to the Discussion section that discusses international law protections for WASH infrastructure.

Revisions from Reviewer #2

In the discussion, I suggest adding 1-2 paragraphs with detailed examples from past conflict zones on the destruction of WASH facilities and subsequent health impacts. This would help to emphasize the public health significance of the study's findings.

Thank you for your helpful feedback. We have added text discussing the public health effects of WASH infrastructure damage in other conflict zones, such as Syria and Ukraine.

It would also be helpful to provide specific details on how to access the data (e.g., URL, retrieval methods) in the methodology section to facilitate replication by other researchers. Additionally, if any software was used to process and analyze the images, please specify it.

We have added text describing how we obtained the raster data and the software we used to process it.

In Figure 4, using completely different colors for the legend might improve readability.

We agree and have revised Figure 4 to be more legible.

Finally, we made two additional changes: an update to the verbiage pertaining to polio (since the vaccination campaign that was underway at the time of initial submission has now been completed) and a removal of one erroneous point from our database that was accidentally included in our initial submission.

Thank you very much for your support, and we look forward to continuing to work with you on the publication of this paper.

Sincerely,

Brian Perlman
Johns Hopkins University
bperlma3@jhu.edu

Shalean M. Collins, PhD
Tulane University
scollin4@tulane.edu

Jamon Van Den Hoek, PhD
Oregon State University
vandenhj@oregonstate.edu
